# Could the Length of the Alkyl Chain Affect the Photodynamic Activity of 5,10,15,20-Tetrakis(1-alkylpyridinium-4-yl)porphyrins?

**DOI:** 10.3390/molecules29061285

**Published:** 2024-03-14

**Authors:** Miryam Chiara Malacarne, Marzia Bruna Gariboldi, Emanuela Marras, Enrico Caruso

**Affiliations:** Department of Biotechnology and Life Sciences (DBSV), University of Insubria, Via J.H. Dunant 3, 21100 Varese, Italy; marzia.gariboldi@uninsubria.it (M.B.G.); emanuela.marras@uninsubria.it (E.M.); enrico.caruso@uninsubria.it (E.C.)

**Keywords:** photodynamic therapy (PDT), tetrakis(*N*-alkylpyridinium-4-yl)porphyrins, tumor cells, cytotoxicity, cellular uptake, cell death mechanisms, ROS

## Abstract

Photodynamic therapy (PDT) is a minimally invasive treatment that uses the combination of a photosensitizing agent (PS) and light to selectively target solid tumors, as well as several non-neoplastic proliferating cell diseases. After systemic administration, PSs are activated by localized irradiation with visible light; in the presence of adequate concentrations of molecular oxygen, this causes the formation of reactive oxygen species (ROS) and subsequent tissue damage. In this study, two series of tetrakis(*N*-alkylpyridinium-4-yl)porphyrins were synthesized, differing in the presence or absence of a zinc ion in the tetrapyrrole nucleus, as well as in the *N*-alkyl chain length (from one to twelve carbon atoms). The compounds were chemically characterized, and their effect on cell viability was evaluated using a panel of three tumor cell lines to determine a possible relationship between photodynamic activity and Zn presence/alkyl chain length. The types of cell death mechanisms involved in the effect of the various PSs were also evaluated. The obtained results indicate that the most effective porphyrin is the Zn-porphyrin, with a pendant made up of eight carbon atoms (**Zn-C8**).

## 1. Introduction

Photodynamic therapy (PDT) is a modern, non-invasive form of therapy used in the treatment of non-oncological diseases and various types of cancer. PDT has been used as an alternative or as an adjuvant to classical oncological therapies for the management of cancers of the esophagus, lung, larynx, cervix, skin, eye, head, and neck [1,2]. However, its use for deep-seated tumors is limited due to insufficient light flux and the risk of damage to superficial tissues [3,4].

**Figure 1:**
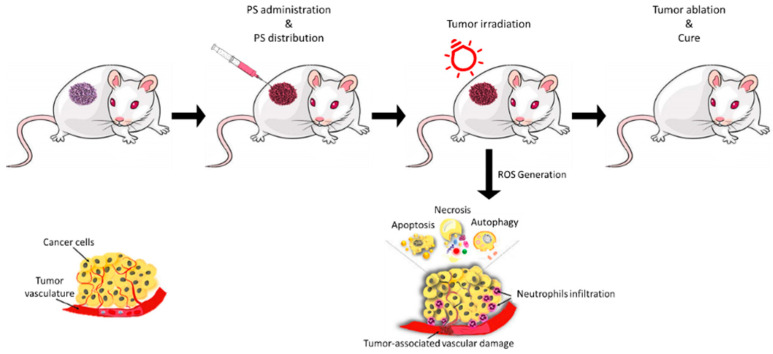
Illustrative scheme of the main phases of photodynamic treatment. Reprinted from Ref. [11].

PDT is based on the local or systemic administration of a photosensitive compound (a photosensitizer, or PS) which accumulates in pathological tissues and, once irradiated with light at specific wavelengths in the presence of molecular oxygen, induces photocytotoxicity reactions in pathological tissues, allowing for their selective destruction (Figure 1) [5,6,7,8].

The absorption of radiation causes the PS to pass from the ground state (S_0_) to the singlet excited state (S_1_). In this state, the PS tends to return to the ground state by emitting fluorescence or by promoting intersystem crossing, which converts the PS to the triplet state (T_1_). From this state, the PS can release energy by interacting with the substrate in the type I reaction, which leads to reactive intermediates, such as hydroxyl radicals and hydrogen peroxide, or it can interact with the molecular oxygen, forming singlet oxygen (^1^O_2_) in the type II reaction. The products obtained from the type I and type II reactions, namely the highly toxic reactive oxygen species (ROS), at the cellular level can generate irreversible damage, progressing to cell death [9,10].

The activation of PDT-induced necrosis, apoptosis, and autophagy depends on the properties and concentrations of PS, the dose of light, and the amount of oxygen present in the tissue. Tissue specificity is made possible by a preferential accumulation of PS in neoplastic tissue rather than in host tissue [12].

The ability to prevent the induction of drug resistance, reduced invasiveness, and the selective distribution of PS are among the advantages of PDT. This last characteristic allows for the repetition of the treatment without incurring side effects and the possibility of directing the light, thanks to the use of endoscopes [13].

Clinical research regarding photodynamic therapy combined with chemotherapy, radiotherapy, and immunotherapy has shown an increase in therapeutic performance compared to that noted for single treatments [4].

The most-used PSs belong to the class of porphyrins, which are colored pigments present in nature as biologically active compounds in living organisms that play important roles in metabolism.

The diversity of porphyrin functions is due, in part, to the variety of metal ions that bind to the core of the ring system [14]. The possibility of providing intersystemic conversion in the triplet excited state allows for their use in photodynamic therapy [15].

Several porphyrin-based PSs, such as porfimer sodium (Photofrin^®^), verteporfin (Visudyne^®^), temoporfin (Foscan^®^), and more recently, padeliporfin (Tookad Soluble^®^), have been approved by the U.S. Food and Drug Administration (FDA) and have entered clinical practice.

Nevertheless, there are still significant challenges in regards to the development of PSs that exhibit improved tumor cell specificity [16,17]. PDT efficacy depends on the photochemical, photobiological, and pharmacokinetic properties of the PS. Hydrophobicity is a desired characteristic, since the solubilization of PS in the membrane bilayer has been highlighted as one of the main factors providing its effectiveness. At the same time, due to hydrophobic interactions, PSs easily form aggregates in aqueous media, thus decreasing their photodynamic effectiveness [18,19]. On the other hand, too much hydrophilicity diminishes its uptake across the cell membrane [20,21,22] and limits the use of the PS in vascular-targeted PDT. Therefore, the balance of the lipo-/hydrophilic character of PSs can be considered one of the most relevant factors for achieving the cellular uptake and effectiveness of PDT [22].

Among the various categories of PS, the most common and widespread is represented by porphyrins which, although presenting some limitations, still play a primary role in the photodynamic field today, as reported in some recent reviews [23,24,25]. Within this category, it may be interesting to consider tetracationic porphyrins, which have been widely used for antimicrobial photodynamic therapy (aPDT) [26,27,28,29], while in the anti-tumor field, they have been used to a much lesser extent [30,31,32]. The reasons for this lie in their excessive hydrophilicity, which identifies these molecules as excellent PSs for aPDT, but makes them less applicable in anti-tumor PDT. To make these molecules more suitable for anti-tumor PDT, it is necessary to modulate the degree of hydrophilicity. In this sense, tetrapyridyl porphyrin could be used as a basic molecule which can be alkylated with chains of various lengths to modulate the degree of amphiphilicity [33,34,35].

Furthermore, porphyrins can be easily metalated, and metals such as zinc are known to increase their ^1^O_2_ production [36]. This characteristic is not always directly linked to a greater photodynamic efficacy [37]; nevertheless, in some works, a greater photodynamic potency of Zn-porphyrins has been observed [32].

Considering the above and that several recent studies have demonstrated that positively charged tetrakis(*N*-alkylpyridinium-4-yl)porphyrins present an interesting anti-tumor photodynamic activity [32,38,39,40,41], the aim of this work was the synthesis and in vitro study of a series of eight tetrakis(*N*-alkylpyridinium-4-yl)porphyrins, four of them as free bases and the others coordinated with a zinc ion. The alkyl chains are characterized by different lengths (1, 4, 8, and 12 carbon atoms) in order to evaluate how much the length of the chain can affect antitumor photodynamic activity. A similar study has already been reported by Pavani, but this study analyzed the photodynamic behavior of only four of the eight molecules used in the present article [33]. Furthermore, in addition to the greater number of molecules studied in this work, additional in-depth chemical–physical and biological studies were also performed. For the eight porphyrins, the degree of lipophilicity, the resistance to photobleaching, and the kinetics of singlet oxygen production were also measured. The photodynamic activity was then assessed on three human tumor cell lines of different tissue origin (i.e., the ovarian carcinoma SKOV3, the colon adenocarcinoma HCT116, and the breast adenocarcinoma MCF7 cell lines) through MTT assay, along with possible differences in cellular uptake. Moreover, the cell death mechanisms involved in the phototoxicity of the studied PSs were also evaluated.

## 2. Results and Discussion

### 2.1. Synthesis and Chemical–Physical Analyses

**Figure 2:**
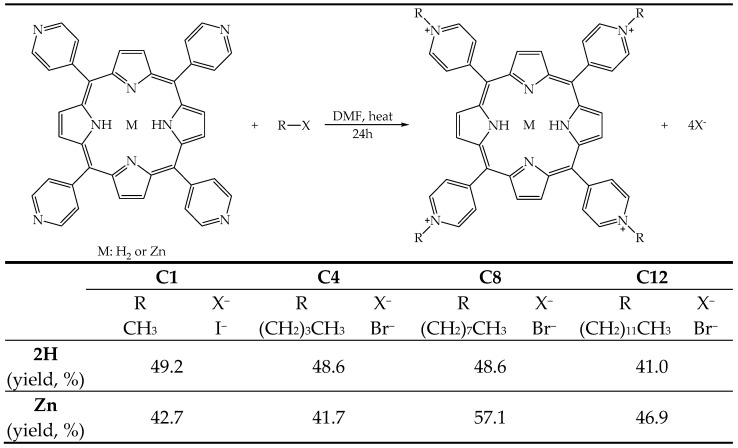
Illustrative scheme of the reaction to obtain the tetrakis(*N*-alkylpyridinium-4-yl)porphyrins, the substituents, and the yield of the eight PSs.

The molecules were obtained by reacting the free base (2H) or metalated (Zn^2+^) 5,10,15,20-tetra(4-pyridyl)porphyrin with an alkyl halide (R-X), with different lengths in the alkyl chain, to introduce four positive charges in the porphyrin nucleus, resulting in pendants of different lengths capable of modulating the amphiphilic character of the molecules, from the most hydrophilic, bearing the methyl pendant (**2H-C1** and **Zn-C1**), to the most lipophilic, bearing the 12-carbon atom pendant (**2H-C12** and **Zn-C12**) (Figure 2), according to procedures reported in the literature [33,42,43,44,45].

All the molecules were recovered by employing precipitation from the reaction environment by adding high quantities of ethyl ether and performing subsequent filtration. The molecules were then characterized by UV-Vis and fluorescence spectroscopy (Table 1), HPLC, and ^1^H NMR.

The absorption spectra show the typical trend for porphyrins [46]. Free-base compounds exhibit a Soret band at 428 nm, which undergoes a slight bathochromic shift of 8 nm with the introduction of the zinc ion. The bathochromic shift is also accompanied by a hyperchromic effect, which is approximately 5% for **Zn-C1**, while it is particularly intense in the case of **Zn-C4**, **Zn-C8**, and **Zn-C12** (30%). The free-base porphyrins present the classic four Q bands, while, as expected, only two remain in the Zn derivatives. However, Zn derivatives show a much higher molar extinction coefficient (ε) in the Q bands compared to that in the free bases due to the interaction between the zinc ion and the π system [45]. The different alkylation does not substantially influence the absorption spectrum [47].

As expected, the fluorescence emission and the fluorescence quantum yield, determined in methanol, showed no substantial differences even if different alkyl pendants were used. The presence of the zinc ion increased the fluorescence quantum yield only for Zn-C8 and Zn-C12. These results were not surprising, as it is reported in the literature that the effects caused by the introduction of zinc ion vary, depending on the structure of the porphyrin [48,49].

All porphyrin derivatives show low fluorescence quantum yields (<0.02); these results agree with those obtained for similar porphyrins [47,50]. Such low overall values indicate that the fluorescence process is not a priority process in the relaxation of the excited singlet state of porphyrins, suggesting that inter-system crossing, which favors the formation of singlet oxygen, can play a very important role in the recovery of the fundamental electronic state.

To evaluate this hypothesis, the single oxygen production quantum yield (Φ_Δ_) was determined (Table 1), which was evaluated through an indirect method that analyzes the decay kinetics of 1,3-diphenylisobenzofuran (DPBF). DPBF shows an absorption peak at 418 nm; however, when the molecule reacts with ^1^O_2_ produced by PS, the absorption peak tends to decrease. The values reported in the table were derived from normalization with respect to **2H-C1**, as it has a Φ_Δ_ value previously reported in the literature [33].

Overall, the values are indicative of a high production of singlet oxygen, confirming the priority role of the ISC in the decay of the excited PS to the fundamental electronic level. Even if the differences are not significant, a slight tendency for Zn-porphyrins to show a greater Φ_Δ_ is observed; on average, this is in agreement with the results in the literature, where it is reported that the coordination with zinc ion favors the ISC process [51]. Regarding the pendants, the PSs with the two longest alkyl chains (C8 and C12) show the highest ^1^O_2_ production in the two series.

The last comment concerns the influence of external iodine atoms, present as counterions in **C1** derivatives, on the effectiveness of the Φ_Δ_. Not directly linked to the tetrapyrrole structure, a secondary heavy-atom effect is caused by these iodine atoms, which might cause a limited increase in the production rate of ^1^O_2_. However, while they are significantly lower compared to those of the C8 and C12 porphyrins, the values observed for these two porphyrins are slightly higher than those noted for the two C4 porphyrins.

**Figure 3:**
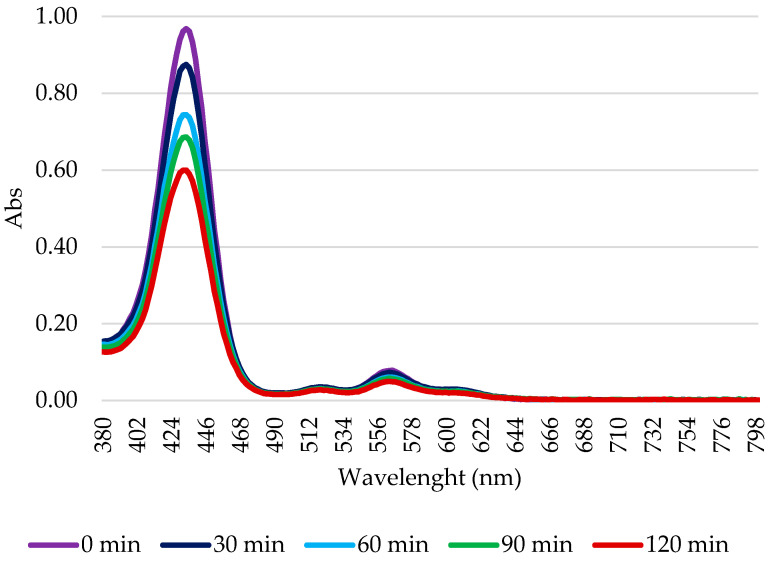
Illustrative scheme of **Zn-C8** photodegradation. A 10 μM solution in 1× PBS was illuminated using a tungsten halogen lamp for 2 h. Every 30 min, a sample was subjected to spectrophotometric analysis. The percentage of photodegradation was calculated as the ratio between the intensity of absorption and the absorption at t_0_.

In in vivo PDT applications, the photodegradation of the PSs plays an important role. Indeed, any PS with high stability can remain highly phototoxic for a prolonged period after the treatment. However, a PS quickly decomposed by light cannot be applied in therapy, as its efficacy could end before the complete treatment of the pathological tissue. Consequently, the photodegradation of the PSs was also evaluated to verify the maintenance of the partial/total integrity of the PS during the irradiation times used in biological protocols. Figure 3 shows an explanatory graph of the gradual decay of the PSs absorption spectrum over time during the 2 h of illumination; it can also be observed that no new peaks appeared.

As can be observed in Table 2, after 2 h of illumination, all the porphyrins maintained an acceptable level of stability. Furthermore, coordination with the zinc ion made the molecules more photo-stable; regardless of the pendant length, the zinc-porphyrins were always more stable than the corresponding free bases. Finally, the data show greater stability for the porphyrins with the shortest pendant (**2H-C1** and **Zn-C1**) compared to that of those with the longest pendant (**2H-C12** and **Zn-C12**).

The length of the alkyl chain proved to be decisive in terms of both photobleaching resistance and ^1^O_2_ production, but in an opposite manner. Considering photobleaching, the porphyrins with shorter alkyl chains were more effectively preserved from photodegradation, while in regards to ^1^O_2_ production, the longer chains compounds provided the best results. The opposite effect observed in these experiments may be partly explained by the method used: photostability is determined in an aqueous medium (PBS) where aggregation is expected, at least for long alkyl-chain porphyrins, while ^1^O_2_ production is determined in an organic solvent, in which no aggregation is observed. However, in both cases, a significantly improved effect in the presence of zinc coordination is observed [52]. Regarding the ^1^O_2_, the result is not surprising, given that it has been observed that metal porphyrins, and in particular, those metalated with zinc, are better ^1^O_2_ producers compared to the same free-base samples [53].

The biodistribution of PS, which influences PDT efficiency and toxicity, is related to several factors, including the route of administration and the distribution between blood and tissues/organs [54]. For intravenous administration, PS should be hydrophilic, with a hydrophobic element to facilitate cell membrane crossing. The evaluation of LogP can estimate the degree of lipophilicity. Although important, this parameter is not a key factor when designing the best PS [55]. In this study, the determination of LogP allows us to define the order of lipophilicity due to the alkylating groups and the possible effect of coordination with the zinc ion.

The octan-1-ol-water partition coefficient (P = [porphyrin]o/[porphyrin]w) of the eight porphyrins was evaluated at 25 °C by stirring equal volumes of water and octan-1-ol containing 40 µM porphyrin for 24 h. The 10-fold dilution with DMF of both the aqueous and the octan-1-ol phase, after partitioning, produces PS solutions with equivalent extinction coefficients, thus allowing a direct evaluation of the PS concentration in both phases. The LogP experimental values for the analyzed compounds are reported in Table 2.

The obtained results confirm that increasing the number of carbon atoms in the N-alkylating chain leads to increased lipophilicity: C1 and C4 molecules exhibited a greater hydrophilic character (greater distribution in water), while the C8 and C12 showed a greater lipophilic character (greater distribution in octan-1-ol). Interestingly, the presence of zinc does not significantly modify the LogP values.

### 2.2. Biological Analyses

**Figure 4:**
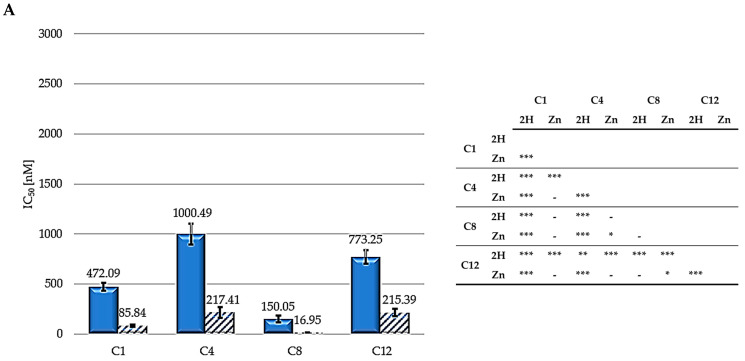

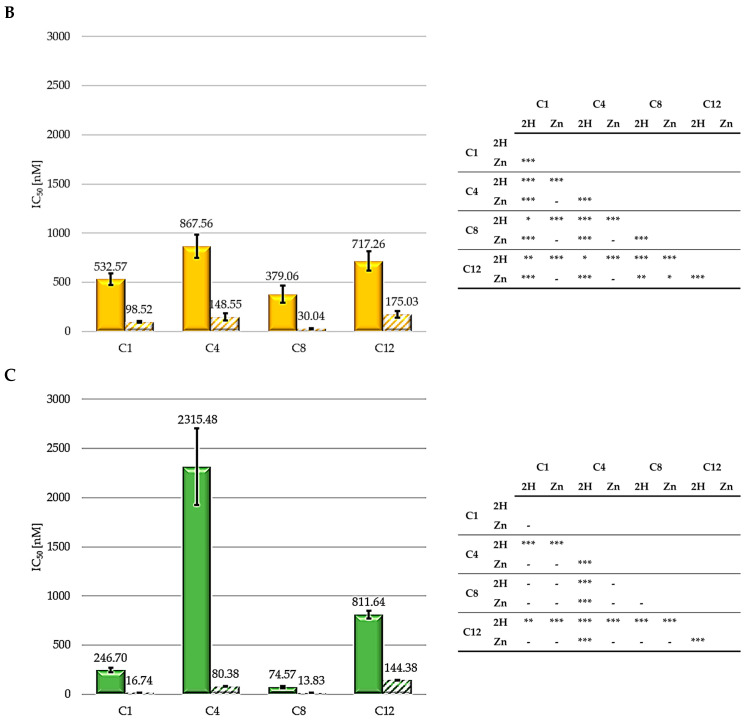
IC_50_ values for HCT116 (**A**), SKOV3 (**B**), and MCF7 (**C**) (**2H**: solid bars; **Zn**: striped bars) and statistical significance. Mean ± SD of five independent experiments. Statistical analyses were performed using two-way ANOVA, followed by Duncan’s post hoc test. *** *p* < 0.001; ** *p* < 0.01; * *p* < 0.05.

The IC_50_ values from the dose—response curves obtained in HCT116 (human colon adenocarcinoma), SKOV3 (human ovarian carcinoma), and MCF7 (human breast) cells following exposure to the different PSs for 24 h, irradiation with a 500 W halogen lamp for 2 h (fluence 158 J/cm^2^), incubation in a drug-free medium for 24 h, and an MTT assay are reported in Figure 4. The intrinsic effects were assessed by omitting the irradiation step, and were observed to be negligible in all cases, although PS concentrations which were 10-fold higher than those used for the PDT experiments were used. Other authors indicated that results obtained with MTT assay could be not sufficient to evaluate photokilling, and a clonogenic assay should be also performed to achieve this aim [56]. Nevertheless, an MTT assay represents a quick and reliable method to evaluate the photodynamic effects of PSs [57].

As shown, zinc-porphyrins exhibit greater activity than their free-base counterparts; this result agrees with those previously reported by Pavani [33]. Regarding the series of metalated porphyrins, the activity trend is **C8** > **C1** > **C4** > **C12**, from the most to the least active. For the free-base series, the **2H-C8** molecule is the most active, followed by **C1**, **C12**, and **C4**. Therefore, taken together, these results indicate a consistent trend for alkyl chains, indicating that the two porphyrins (2H and Zn) exhibiting a 8-carbon alkyl pendants are the most effective. C1 always ranked second in the two series (Zn and 2H) in all three cell lines. Contrastingly, C4 and C12 exhibited the worst results.

The significantly higher photodynamic activity observed following the treatment of HCT116, SKOV3, and MCF7 cells with the Zn-porphyrins, compared to that observed for treatment with the 2H-porphyrins, is consistent with the higher singlet oxygen production observed for Zn-porphyrins. In addition, the reported results seem to indicate that the 8-carbon alkyl groups provide the molecules with the characteristics suitable for achieving superior photodynamic effectiveness.

To determine whether the different activity levels were strictly linked to a difference in PS uptake, a flow cytometry experiment was conducted.

**Figure 5:**
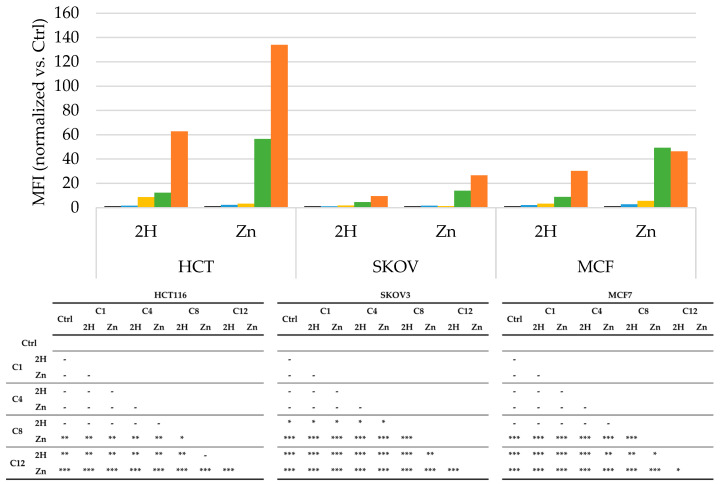
Uptake, obtained by cytometric analysis, expressed as mean fluorescence intensity (MFI) values obtained after treatment with 100 nM of each PS, and the normalized vs respective controls, for HCT116, SKOV3, and MCF7 cells (**Ctrl**: black; **C1**: blue; **C4**: yellow; **C8**: green; **C12**: orange), along with the statistical significance. Mean ± SD of five independent experiments. Statistical analyses were performed using two-way ANOVA, followed by Duncan’s post hoc test. *** *p* < 0.001; ** *p* < 0.01; * *p* < 0.05.

As reported in Figure 5, in both series, the C8 and C12 molecules were the most frequently taken up in the examined cell lines. Overall, the order relating to cellular absorption, C12 > C8 > C4 > C1, follows exactly the same order of lipophilicity determined using LogP. This result is undoubtedly related to the greater similarity to the cell membrane in terms of lipophilicity and is in agreement with what was reported by Azzeddine for a series of Zn(II) tetrakis(*N*-alkylpyridinium-4-yl)porphyrins [41]. In agreement with LogP data, except for C4, Zn-PSs showed better absorption compared to that of the free-base molecules.

However, the uptake results are not in agreement with the results for photodynamic efficacy. This result is not surprising, since has been reported that the effectiveness of a PS strongly depends on its subcellular localization, which has been not evaluated in the present study. In addition, in a previous study by our group, we observed that the photodynamic activity is not directly correlated with the LogP value [37], and other authors have observed that there is not always a direct correlation between the uptake and the photodynamic activity [58,59].

To detect the intracellular production of ROS, cells were treated with PS, at IC_50_, for 24 h and irradiated for 2 h before incubation with DCHF-DA. Following the detection of the dichlorofluorescein fluorescence by a fluorescence microscope, the ROS production rate was measured using ImageJ software (1.49V). Recently, it has been reported that adding DCHF-DA after the irradiation step can result in the underestimation of ROS levels, due to their short half-life. Thus, it is possible that we only evaluated the presence of long-lasting ROS, namely H_2_O_2_, or ROS generated following irradiation, such as lipid peroxides [60].

**Figure 6:**
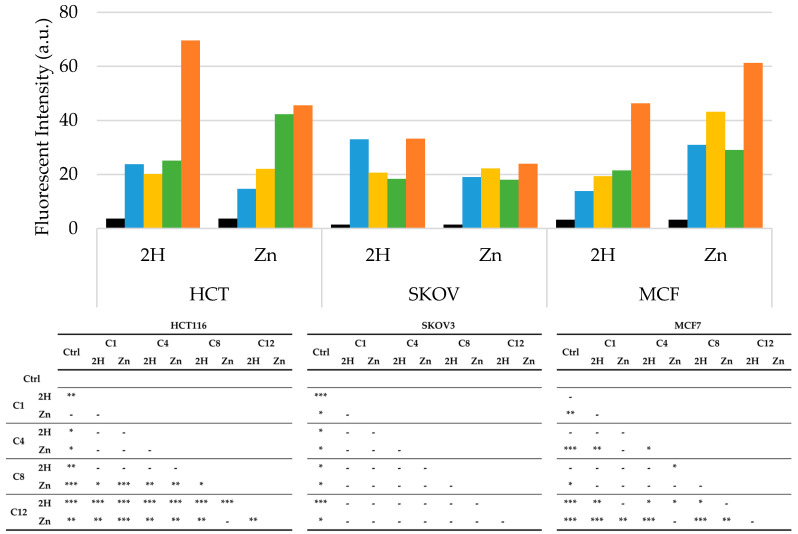
Arbitrary fluorescence units of ROS in HCT116, SKOV3, and MCF7 cells after PDT treatment (**Ctrl**: black; **C1**: blue; **C4**: yellow; **C8**: green; **C12**: orange), along with statistical significance. In the control samples, PS treatment was omitted. Mean ± SD of five independent experiments. Statistical analysis was performed using two-way ANOVA, followed by Duncan’s post hoc test. *** *p* < 0.001; ** *p* < 0.01; * *p* < 0.05.

Figure 6 shows that all porphyrins induced a significant increase in ROS levels over those of the control in all cell lines. Specifically, the SKOV3 cells showed the lowest ROS levels in response to PSs treatment, and no differences were observed between Zn- and base-free porphyrins when compared to the other two cell lines. In HCT116, the two C12 porphyrins showed the highest ROS levels, while a specific trend was not observed between the 2H and Zn-porphyrins.

It can be observed that the highest ROS production occurred in the MCF7 cell line, which was also the most sensitive to PDT. Furthermore, in this cell line, the Zn-porphyrins induced higher ROS levels than those of their 2H counterparts, as previously demonstrated by other authors [61].

Overall, the ROS results, as in the case of those for the LogP, do not exhaustively explain the different photodynamic effectiveness observed. This is not unexpected because many parameters are known to correlate PS structure with photodynamic activity [37].

It is generally recognized that through the production of ROS and/or ^1^O_2_, photochemical reactions can trigger one or more cell death mechanisms, mainly apoptosis and necrosis, as well as autophagy, which lead directly to tumor destruction [62,63,64].

**Figure 7:**
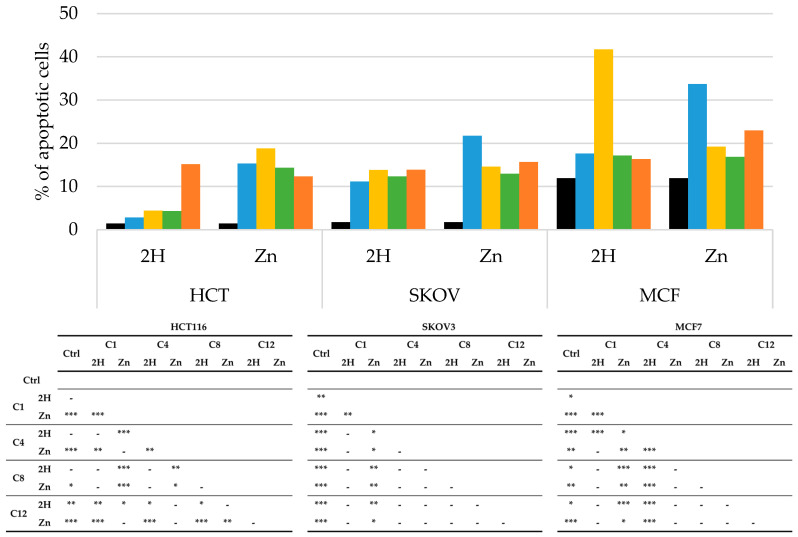
Percentage of apoptotic cells, obtained by flow cytometric analysis, after PDT treatment on HCT116, SKOV3, and MCF7 cells (**Ctrl**: black; **C1**: blue; **C4**: yellow, **C8**: green; **C12**: orange), along with statistical significance. Mean ± SD of five independent experiments. Statistical analyses were performed using two-way ANOVA, followed by Duncan’s post hoc test. *** *p* < 0.001; ** *p* < 0.01; * *p* < 0.05.

**Figure 8:**
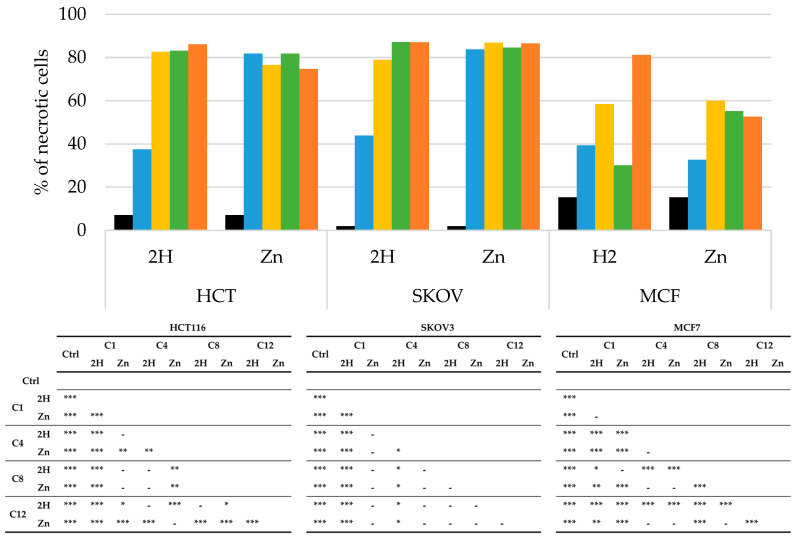
Percentage of necrotic cells, obtained by flow cytometric, after PDT treatment on HCT116, SKOV3, and MCF7 cells (**Ctrl**: black; **C1**: blue; **C4**: yellow; **C8**: green; **C12**: orange) and statistical significance. Mean ± SD of 5 independent experiments. Statistical analyses were performed using two-way ANOVA, followed by Duncan’s post hoc test. *** *p* < 0.001; ** *p* < 0.01; * *p* < 0.05.

PDT-induced apoptosis or necrosis was evaluated through flow cytometric analysis using PI as a probe, and the obtained results are reported in Figure 7 and Figure 8.

In the absence of irradiation, none of the compounds triggered apoptotic or necrotic responses, compared to the results for the control.

As observed in Figure 7, nearly all the molecules increased the percentage of apoptotic cells compared to those in the control. However, apoptosis did not seem to be the main mechanism of cell death in any of the cell lines. As a matter of fact, in the HCT116 and SKOV3 cells, treatment with all of the experimental compounds caused less than 20% apoptosis, while in the MCF7 cells, slightly higher percentages of apoptosis were observed for treatment with **Zn-C1** and **2H-C4**; nevertheless, the levels were always below 50%.

As reported in Figure 8, all the PSs analyzed induced necrotic cell death.

In the HCT116 and SKOV3 cells, all of the zinc compounds directed the cells toward necrosis (80%); this behavior was also observed for the free-base compounds, except for **2H-C1**, which, in both cell lines, induced around 40% necrosis. As previously observed for apoptosis, the MCF7 cells responded differently from the others. In this cell line, all the compounds induced overall lower levels of necrosis.

Generally, we observed that in the HCT116 and SKOV3 cell lines, the main mechanism of cell death was necrosis, with only a small contribution by apoptosis. On the other hand, in the MCF7 cells, apoptosis and necrosis appear to contribute equally to the photodynamic effects of PSs.

In recent years, several studies have shown that PDT can trigger autophagy, a catabolic degradation process able to mediate the turnover and recycling of cytoplasmic components [65,66]. Although the relationship between autophagy and cell death in PDT is still under discussion in the literature, with authors showing that an increase in autophagy upon PDT may lead either to cytoprotection or cytotoxicity in a cell-dependent manner [67], several studies have determined autophagy to be responsible for the cell damage generated by many PSs, showing that autophagic cell death may occur together with apoptosis or necrosis [68,69].

To assess the degree of autophagy induced by the studied compounds, the total cell lysates of the treated cells were subjected to Western blot analysis to detect the presence of LC3-II, a known marker for autophagy.

**Figure 9:**
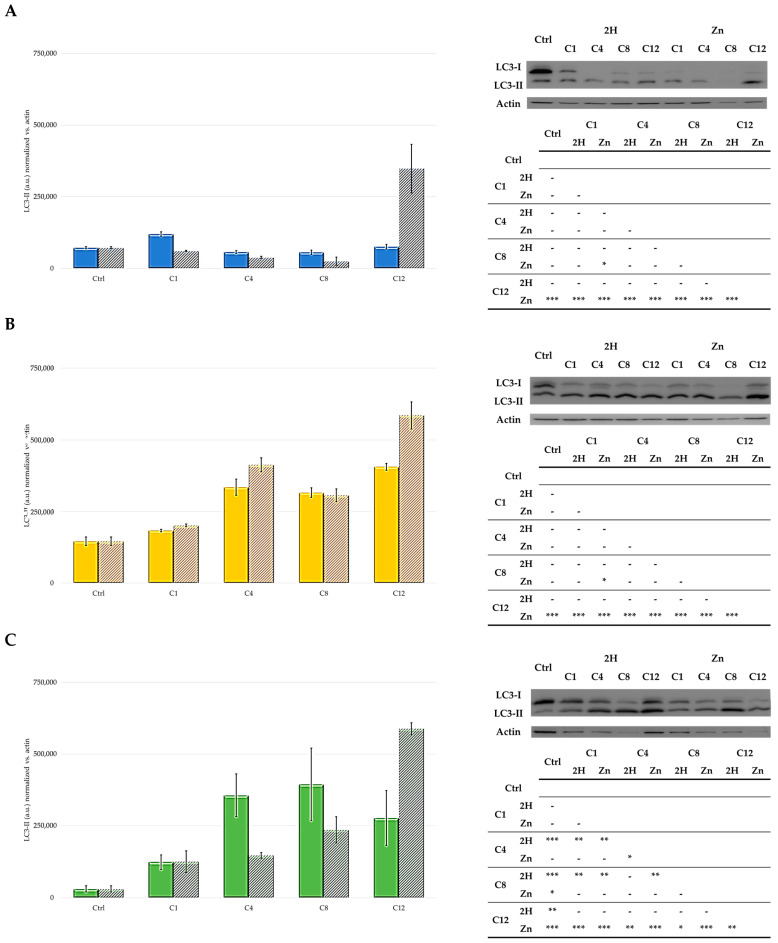
Western blot and densitometric analysis of LC3-II levels in HCT116 (**A**), SKOV3 (**B**), and MCF7 (**C**) cells (**2H**: solid bars; **Zn**: striped bars). In control samples, PS treatment was omitted, and the mean ± SD of three independent experiments was included. Statistical analyses were performed using two-way ANOVA, followed by Duncan’s post hoc test. *** *p* < 0.001; ** *p* < 0.01; * *p* < 0.05.

Our data showed that cell-type differences were present in the autophagic response to the treatment. In particular, the MCF7 and SKOV3 cell lines responded to the treatment with the 2H- and Zn-porphyrins by activating autophagy, despite the fact that there was not a clear trend related to the type of PSs (2H or Zn) (Figure 9). On the contrary, in the HCT116 cells, high levels of the autophagic marker LC3-II were only observed following treatment with Zn-C12. However, the specific role of this process in the cytoprotective or cytotoxic effect of the studied PSs was not evaluated in the present study.

In conclusion, compared to Pavani’s work [33], in this study, we considered a wider range of compounds to confirm the effects of zinc coordination and to better understand the effects of the alkyl chain length on the chemical-physical properties, photodynamic activity, and cellular uptake of the PSs. Furthermore, the effects of PSs were evaluated on a larger number of cancer cell lines, originating from different tissue types.

Results obtained show a stronger anti-tumor PDT-related effect for the Zn-porphyrin series compared to the counterpart (2H). Furthermore, the analysis of the results obtained from two series in the different cell lines indicates that C8 is the most interesting molecule among those included in the study. This is related to the combination of favorable chemical–physical characteristics observed, which are all required and desired for a PS candidate. In particular, C8 showed the best PDT-related anti-tumor effect, along with good photostability, the high production of singlet oxygen, and a fair cellular uptake.

The reported results indicate that Zn-metalated porphyrins, mainly C8 porphyrins, could represent potential new PSs for anticancer PDT and therefore, are worthy of further studies to better understand their mechanisms of action.

## 3. Materials and Methods

### 3.1. Chemicals and Experimental Instruments

The UV-Vis absorption spectrum is measured using a Cary 60 UV-Vis Spectrophotometer (Agilent, Santa Clara, CA, USA).

Steady-state fluorescence measurements are performed using a Jasco model FP-750 spectrofluorometer (Jasco Inc., Tokyo, Japan).

For all compounds, 1 mM stock solutions are prepared in DMSO, once the necessary analyses are carried out to confirm their composition.

For the chemical analyses that required the use of light sources, a green LED light source or a 500 W tungsten white halogen lamp is used. The green LED light source (526 ± 46 nm) consists of 12 LEDs, 1 W each, for a total of 12 W. The control unit connects to the lamp via an RJ45 connection and is powered by a constant current of 350 mA. The source has an irradiance equal to 3.036 mW/cm^2^, equal to 5.45 J/cm^2^ of fluence for 30 min of illumination.

The irradiation device using a tungsten lamp is placed above the area to be irradiated at a distance to produce a homogeneous irradiation area. For this type of lamp, a cooling device is required to avoid overheating; therefore, a flowing water filter was placed between the light and the irradiation area. The lamp has an irradiance of 22 mW/cm^2^, equal to 158 J/cm^2^ of fluence for 2 h of irradiation.

### 3.2. Synthesis: General Procedure for the Preparation of 5,10,15,20-Tetrakis(1-alkylpyridinium-4-yl)porphyrins

The free-base porphyrins, **2H-C1**, **2H-C4**, **2H-C8**, and **2H-C12**, and the corresponding Zn metalated samples, **Zn-C1**, **Zn-C4**, **Zn-C8**, and **Zn-C12**, were synthetized as reported in the literature [33,42,43,44,45].

### 3.3. Singlet Oxygen Generation

To evaluate the amount of ^1^O_2_ produced by the compounds, a solution of 8 mL of isopropanol, 50 μM of 1,3-diphenylisobenzofuran (DPBF), and 2.5 μM of PS is prepared. A total of 2 mL of this solution is transferred to a quartz cuvette and irradiated from above for 30 min using a green LED (12 led at 1 W, for a total of 12 W, an RJ45 connection, a constant current of 350 mA, and an irradiance of 1.9 mW/cm^2^ for the green LED and 9.24 mW/cm^2^ for the blue LED, respectively), as reported in literature [70]. A sample containing only DPBF and isopropanol is used as a negative control. The decrease in DPBF absorbance at 410 nm was observed using a Varian Cary 50 Scan UV Visible Spectrophotometer (Agilent, Santa Clara, CA, USA) (every 20 s for 20 min). The obtained absorption spectrum was used to calculate ^1^O_2_, using the singlet oxygen production of **2H-C1** as reference.

### 3.4. Photobleaching

A 10 μM solution is prepared in 1X PBS for each sample. The solutions thus obtained are illuminated using the tungsten halogen lamp for 2 h. At set times, samples are taken and subjected to spectrophotometric analysis. The percentage of photodegradation is calculated as the ratio between the intensity of absorption and the absorption at t_0_.

### 3.5. Partition Coefficient Measurements

The octan-1-ol-water partition coefficient (P) of the eight porphyrins was determined at 25 °C in equal volumes of pre-equilibrated water (milliQ, 3 mL) and octan-1-ol (3 mL), as previously reported [71]. An equal volume of octan-1-ol solution of porphyrin (40 µM) and water was stirred for 8 h. Next, the two previously separated phases were diluted ten times with DMF, and the solutions were analyzed using UV-Vis spectroscopy. LogP was calculated, as reported in Equation (1):

(1) $$LogP=\frac{{\left\lfloor Porphyrin\right\rfloor }_{octan-1-ol}}{{\left\lfloor Porphyrin\right\rfloor }_{H_{2}O}}$$

### 3.6. Cell Culture

The human colon carcinoma HCT116, the ovarian cancer SKOV3, and the breast cancer cell line MCF7 were obtained from the American Type Culture Collection (Rockville, MD, USA).

The HCT116, the SKOV3, and the MCF7 cell lines are maintained in Dulbecco’s Modified Eagle Medium (DMEM), Dulbecco’s Modified Eagle Medium/Nutrient Mixture F-12 (DMEM/F12), and Roswell Park Memorial Institute 1640 (RPMI 1640), respectively, all supplied by Euroclone (Milan, Italy). Culture media are supplemented with 10% fetal bovine serum, 1% glutamine, 0.5% penicillin-streptomycin, and 0.25% amphotericin B (all supplied by Merck, Darmstadt, Germany).

The cells are maintained under standard culture conditions at 37 °C in a humidified atmosphere at 5% CO_2_.

### 3.7. Photodynamic Effects

The photodynamic effects of the studied compounds are evaluated using the MTT ([3-(4,5-dimethylthiazol-2-yl)-2,5-diphenyltetrazolium bromide]) assay [72].

The protocol [73] stipulates cells sowing in a 96-well plate (HCT116 and SKOV3: 2.5 × 10^3^ cells/well, MCF7: 3.5 × 10^3^ cells/well) and subsequent growth for 48 h. After this period, the cells are treated with 100 μL of porphyrin solutions at different concentrations (test concentrations ranging from 0.1 to 10,000 nM). The solutions used for the treatment are prepared by suitably diluting the stock solution (1 mM in DMSO) in the culture medium. The dilutions are obtained to achieve a non-toxic concentration of organic solvent (0.1%) in the medium used for the treatment. In the control samples, treatment with PS is omitted. After 24 h, the medium containing PS is replaced with 1× PBS, and the cells are irradiated for 2 h with a 500 W white tungsten halogen lamp. After irradiation, the PBS is replaced with the PS-free medium. After 24 h, MTT (final concentration 0.4 mg/mL) is added to each well and allowed to act for 3 h at 37 °C. The formazan crystals, formed in vital cells because of MTT metabolism, are dissolved in DMSO. The Infinite^®^ 200 PRO (Tecan, Bio-Rad Inc., Hercules, CA, USA) plate reader, with a 590 nm filter, is used to read the cell survival data. The data obtained are analyzed by non-linear regression using GraphPad PRISM 9.2.0 software (GraphPad Software Inc., San Diego, CA, USA); this analysis allows us to obtain the values of IC_50_.

The possible intrinsic effects of the compounds are evaluated on cell cultures in which the illumination step is omitted, and which were treated with concentrations ten times higher than the maximum concentration used in the assay employing the illumination step.

### 3.8. Flow Cytometric Analyses

Flow cytometric analyses are conducted using FACScalibur (Becton Dickinson, Franklin Lakes, NJ, USA) flow cytometer, equipped with a 15 mW, 488 nm, air-cooled argon laser, and the data are analyzed using Cell QuestPRO V6.0 software (Becton Dickinson, Franklin Lakes, NJ, USA).

#### 3.8.1. Intracellular Uptake

To evaluate PS uptake, the cells are seeded on a 12-well plate (7.0 × 10^4^ cells/well) and exposed to the compounds (100 nM) for 24 h. In this experiment, the cells are not irradiated. After exposure, the cells are detached by trypsinization, washed in ice-cold PBS, resuspended in PBS, and analyzed.

For all cytometric analyses, the PS treatment is omitted in the control samples.

Absorption is quantified in arbitrary units based on mean fluorescence intensity (MFI) by collecting PS fluorescence using a 575 nm band-pass filter [73].

#### 3.8.2. Apoptosis and Necrosis

The assessment of the ability of the compounds to induce death through apoptosis or necrosis is evaluated using a flow cytometric analysis.

The cells are seeded on a 12-well plate (7.0 × 10^4^ cells/well) and left to grow for 48 h. the cells are then treated with PS at the respective IC_50_ values for 24 h and are subsequently irradiated for 2 h (500 W white tungsten halogen lamp) with PBS. After irradiation, the cells are again placed in a Ps-free medium and left in the incubator for 24 h.

To evaluate the percentage of apoptotic cells at the end of the treatment, the cells are collected, washed twice in PBS, and fixed in 70% EtOH at −20 °C for at least 30 min. Following further washing in PBS, the cells are resuspended in a PBS solution containing propidium iodide (PI) (50 μg/mL) and RNase A (30 U/mL).

For the evaluation of necrotic cells, the step of fixation with EtOH is omitted.

For both analyses in the control samples, treatment with PS is omitted.

The fluorescent emission of PI is collected using a 575 nm band-pass filter, and the percentage of apoptotic cells in each sample is determined based on the sub-G1 peaks detected in the single-parameter histograms acquired in log mode [74].

### 3.9. Determination of Intracellular ROS

The 2′,7′-dichlorodihydrofluorescein diacetate fluorogenic probe (DCHF-DA, InVitrogen Molecular Probes, ThermoFischer Scientific, Waltham, MA, USA) is used to evaluate the intracellular generation of ROS. Once internalized by the cells, DCHF-DA is cleaved by intracellular esterases to release the corresponding dichlorodihydrofluorescein derivative (non-fluorescent), which is readily oxidized by ROS to dichlorofluorescein (DCF) [75,76,77,78].

The cells are seeded on a 12-well plate (7.0 × 10^4^ cell/well), treated with PS at the respective IC_50_ for 24 h, followed by 2 h of irradiation (500 W white tungsten halogen lamp) in PS-free PBS. After irradiation, the cells are treated with 10 μM of DCFH-DA in each well. The cells are then incubated in the dark at 37 °C for 30 min. Cell images are acquired using a camera connected to an Olympus IX81 microscope (Olympus Life Science, Waltham, MA, USA). Fluorescence microscopy was used to detect the production of ROS (Ex: 488 nm; Em: 520 nm) [79,80]. For the control samples, treatment with PS is omitted. The ROS production rate, expressed as arbitrary fluorescence units, was measured using ImageJ software [81,82].

### 3.10. Western Blot for Autophagy Detection

The induction of autophagy by the compounds under analysis is evaluated by Western blot analysis; to do this, a rabbit polyclonal antibody directed against the autophagosomal marker LC3-I/II is used.

The 1.0 × 10^6^ cells are seeded in cell culture flasks and left to grow for 48 h, after which the cells are treated with the compounds at the respective IC_50_ values. After 24 h, the cells are illuminated for 2 h (500 W white tungsten halogen lamp) in 1× PBS. For the control samples, treatment with PS is omitted.

After irradiation, the cells are placed in drug-free medium for 24 h. At the end of this time, the cells are lysed with a buffer containing NaCl (120 mM), NaF (25 mM), EDTA (5 mM), EGTA (6 mM), sodium pyrophosphate (25 mM in TBS 20 mM, pH 7.4), PMSF (2 mM), Na_3_VO_4_ (1 mM), phenylarsine oxide (1 mM), 1% *v*/*v* NP-40, and 10% *v*/*v* Protease Inhibitor Cocktail. The protein concentration in each lysate is determined using the BCA test.

A total of 30 μg of proteins per sample are loaded onto polyacrylamide gel (15%) and separated under denaturation conditions. The obtained protein bands are transferred onto Hybond-P (Millipore, Burlington, MA, USA) membranes to perform Western blot analysis. The bands are visualized by G-box (Syngene, Chemi-XT4, Cambridge, UK), using peroxidase-conjugated secondary anti-rabbit antibodies (for LC-3I/II) (Merck, Darmstadt, Germany) and Westar Supernova substrate (Cyanagen, Bologna, Italy).

To check for the equal loading of samples, the blot is reanalyzed with mouse monoclonal anti-actin antibody (Santa Cruz Biotechnology, Inc., Dallas, TX, USA), and the bands are visualized by G-box, following incubation with a secondary anti-mouse antibody (Merck) and the Westar Supernova substrate.

### 3.11. Statistical Analyses

The statistical analyses of the data obtained from the various experiments (at least three independent tests) are performed by two-way ANOVA, followed by Duncan’s post hoc test. The ANOVA test is performed, considering data with a *p*-value less than 0.05 as significant. Data normality is tested using the Shapiro–Wilk test [83].

## Figures and Tables

**Table 1:** Absorption band of the UV-Vis spectra in methanol, along with the relative extinction coefficient ε (M^−1^ cm^−1^), fluorescence quantum yield (Φ_Δ_) and singlet oxygen quantum yield (Φ_Δ_).

		Soret Band ^a^ (nm)	Q Bands ^a^ (nm)				Φ_f_ ^b^	Φ_Δ_
**2H**	**C1**	428 (ε = 1.53 × 10^5^)	515 (ε = 1.4 × 10^4^)	550 (ε = 4.5 × 10^3^)	590 (ε = 4.5 × 10^3^)	645 (ε = 1200)	0.012 (645 nm)	1.00
	**C4**	428 (ε = 1.41 × 10^5^)	515 (ε = 1.3 × 10^4^)	550 (ε = 3.9 × 10^3^)	590 (ε = 4.3 × 10^3^)	645 (ε = 1100)	0.010 (650 nm)	0.92
	**C8**	428 (ε = 1.74 × 10^5^)	515 (ε = 1.3 × 10^4^)	550 (ε = 3.6 × 10^3^)	590 (ε = 4.3 × 10^3^)	645 (ε = 1400)	0.013 (650 nm)	1.42
	**C12**	428 (ε = 1.58 × 10^5^)	515 (ε = 1.3 × 10^4^)	550 (ε = 4.1 × 10^3^)	590 (ε = 4.4 × 10^3^)	645 (ε = 1300)	0.014 (645 nm)	1.77
**Zn**	**C1**	436 (ε = 1.60 × 10^5^)		565 (ε = 1.1 × 10^4^)		610 (ε = 2900)	0.011 (645 nm)	1.22
	**C4**	436 (ε = 1.76 × 10^5^)		565 (ε = 1.3 × 10^4^)		610 (ε = 2300)	0.010 (645 nm)	0.94
	**C8**	436 (ε = 2.35 × 10^5^)		565 (ε = 2.2 × 10^4^)		610 (ε = 4600)	0.018 (650 nm)	1.64
	**C12**	436 (ε = 2.01 × 10^5^)		565 (ε = 1.9 × 10^4^)		610 (ε = 4400)	0.017 (650 nm)	1.78

^a^ 10^−4^ M in methanol, ε = M^−1^ cm^−1^. ^b^ Excitation at the Soret band, emission > 630; 10^−6^ M in methanol. Reference compound—fluorescein.

**Table 2:** Percentage of photostability after 1 h and 2 h of irradiation with a 500 W halogen white light (fluence 158 J/cm^2^)and partition coefficient of each PS.

		% of Photostability		LogP
		1 h	2 h	
**2H**	**C1**	83.80	69.50	−0.64
	**C4**	87.20	40.50	−0.37
	**C8**	75.00	35.70	0.15
	**C12**	55.10	15.00	0.30
**Zn**	**C1**	88.80	74.00	−0.87
	**C4**	68.90	53.30	−0.46
	**C8**	65.20	44.00	0.20
	**C12**	45.40	20.40	0.37

## Data Availability

Data are contained within the article.
